# Unlocking the dual role of LSD1 in tumor immunity: innate and adaptive pathways

**DOI:** 10.7150/thno.102037

**Published:** 2024-10-21

**Authors:** Yu Zhang, Ningjie Guo, Haoyi Zhu, Mengyang Liu, Jiahui Hao, Shoukai Wang, Ting Guo, MAA Mamun, Jingru Pang, Qi Liu, Yichao Zheng, Hongmin Liu, Pilei Si, Lijuan Zhao

**Affiliations:** 1 State Key Laboratory of Esophageal Cancer Prevention & Treatment; Key Laboratory of Advanced Drug Preparation Technologies, Ministry of Education of China; Key Laboratory of Henan Province for Drug Quality and Evaluation, Henan Province; School of Pharmaceutical Sciences; Academy of Medical Sciences; Tianjian Laboratory of Advanced Biomedical Sciences; Zhengzhou University, Zhengzhou, Henan 450001, China.; 2 Department of Breast Surgery, Henan Provincial People's Hospital, People's Hospital of Zhengzhou University, People's Hospital of Henan University; Henan Provincial Engineering Research Center of Breast Cancer Precise Prevention and Treatment, Zhengzhou, Henan 450003, China.; 3 XNA platform, School of Pharmaceutical Sciences, Zhengzhou University, Zhengzhou, Henan 450001, China.; 4 School of Medicine, Taizhou University, Taizhou, Zhejiang 318000, China.

**Keywords:** LSD1, Tumor microenvironment, Immune checkpoint blockade, Immune cell, Cancer immunotherapy

## Abstract

The roles of innate and adaptive immunity are crucial in both the development of cancer and its response to treatment. Numerous studies have demonstrated that histone lysine-specific demethylase 1 (LSD1) is overexpressed in various cancers. Elevated levels of LSD1 intricately modulate immune checkpoints, the function of immune cells, and the expression of immunomodulators, impacting both innate and adaptive immunity. Moreover, compelling evidence suggests that inhibiting LSD1 enhances tumor immunity, suppresses tumor growth, and improves the effectiveness of immunotherapy. However, a comprehensive classification of LSD1's role in both innate and adaptive immunity is lacking. In this review, we outline the role of LSD1 in tumor immunity in terms of both innate and adaptive immunity, summarizing the mechanisms associated with LSD1-mediated tumor immunity and its potential regulatory capacity in tumor immune escape. Finally, we summarize the research status of LSD1 inhibitors in tumor immunotherapy, which be valuable for promoting the development of effective LSD1-targeted agents used as combination immunotherapy drugs.

## 1. Introduction

The human body maintains health through innate and adaptive immunity, which work to recognize and eliminate damaged and tumor cells. In cancer, both systems play crucial roles 1. Adaptive immunity, primarily mediated by B and T cells, is central to the fight against cancer and involves several key steps: antigen recognition, clonal expansion, the effector phase, and immune memory 2, 3. On the other hand, the innate immune system is activated by sensing tissue damage and recruiting cells to the affected areas. For example, macrophages capture and digest diseased cells through phagocytosis, while natural killer (NK) cells can recognize and kill infected or cancerous cells, suppressing tumor development without the need for antigen presentation 2-4. The tumor microenvironment (TME) is a complex ecosystem with both pro-tumor and anti-tumor tendencies. It comprises innate immune cells, adaptive immune cells, immunosuppressive cells, interstitial tissues, microvessels, and various cytokines and chemokines 5-10. Tumor cells typically create an immunosuppressive environment by recruiting immunosuppressive cells 11, 12, secreting factors that suppress the immune response 13-16, overexpressing antigens like PD-L1 17, 18, exhibiting low major histocompatibility complex (MHC) 19, 20, or releasing tumor exosomes to sustain their survival and progression 21, 22.

LSD1, also known as KDM1A, is a crucial epigenetic target involved in various physiological and pathological processes through its demethylase activity on both histone and non-histone substrates 23-25. Numerous studies have shown that LSD1 is highly expressed in many cancers, including gastric cancer 26, squamous cell carcinoma 27, hepatocellular carcinoma 28, prostate cancer 29, and breast cancer 30. High LSD1 expression is generally associated with poor prognosis. Currently, several LSD1 inhibitors are in clinical trials and show considerable potential in cancer treatment 31, 32. Recent research has highlighted the important role of LSD1 in tumor immunity. Abnormally high expression of LSD1 influences tumor immunity in multiple cancers by regulating immune checkpoints 33-35, antigen presentation 36, 37, and other pathways 38. Consequently, LSD1 is a potential target for modulating the TME, offering significant research value and application potential in tumor immunotherapy. Inhibiting LSD1 expression or activity can alter the immunosuppressive characteristics of the TME and enhance the antitumor immune response, ultimately improving the efficacy of tumor immunotherapy.

In this review, the biological function of LSD1 and its implications in immunotherapy are elucidated from the perspectives of innate and adaptive immunity.

## 2. LSD1 and cancer

LSD1, a member of the flavin adenine dinucleotide-dependent amine oxidase superfamily, was the first histone demethylase to be identified 39. It interacts with the nuclear REST corepressor 1 (CoREST) transcriptional blocking complex [40]and histone deacetylases 1 and 2 (HDAC1/2) 41. In complex with CoREST and the nucleosome remodeling and deacetylase, LSD1 removes methyl groups from monomethyl and dimethyl histone H3 lysine 4 (H3K4me1/me2), leading to transcriptional repression 39, 42-45. Beyond histones, LSD1 targets several non-histone proteins, including DNA methyltransferase 1 (DNMT1), E2F transcription factor 1 (E2F1), tumor suppressor p53, transcription signal transducer and activator of transcription 3 (STAT3), and hypoxia-inducible factor-1 (HIF-1α) 24. Through the demethylation of these substrates, LSD1 is involved in various cellular processes, such as cell proliferation and differentiation 46, senescence 47, epithelial-mesenchymal transition 48, chromatin regulation 42, angiogenesis 49, cancer stem cell regulation 50, glycolysis and mitochondrial metabolism 28.

There is growing evidence that abnormal expression of LSD1 in tumors contributes to poor immunotherapy outcomes 51-56. Inhibiting LSD1 expression and transcriptional activity presents a promising new avenue for the immunotherapy of malignant tumors 57, 58. LSD1 mediates tumor immunity through various mechanisms, such as regulating autophagy-related gene expression to mediate tumor-associated autophagy 59, 60, affecting immune checkpoint protein expression 34, 61, upregulating immunosuppressive molecules 62, 63, reducing tumor antigen presentation 36, 37, and interfering with hypoxia-mediated tumor physiology and pathogenesis 64, 65. The elucidation of LSD1's molecular mechanisms in tumor immune escape underscores its potential as a target for tumor immunotherapy. The following sections will explore the intricate roles and molecular mechanisms of LSD1 in both innate and adaptive immunity.

## 3. LSD1 regulates innate immunity

Innate immunity is the first line of defense against invading pathogens and disturbances in homeostasis. Key players in innate immunity include cells derived from bone marrow precursors, such as NK cells, macrophages, and dendritic cells (DCs). These cells are essential for resisting and eliminating pathogenic microorganisms 66. In the early stages of cancer, the innate immune system can recognize and eliminate tumor cells. However, as the tumor develops, the TME becomes immunosuppressive, allowing tumor cells to escape immune surveillance. In this context, innate immune cells can become significant factors promoting tumor growth and metastasis 67. Studies have shown that LSD1 can affect the anti-tumor activity of innate immune cells, either directly or indirectly. The following sections will discuss the role of LSD1 in innate immunity, focusing on its effects on different cell types.

### 3.1 Regulation of NK cells

**Figure 1:**
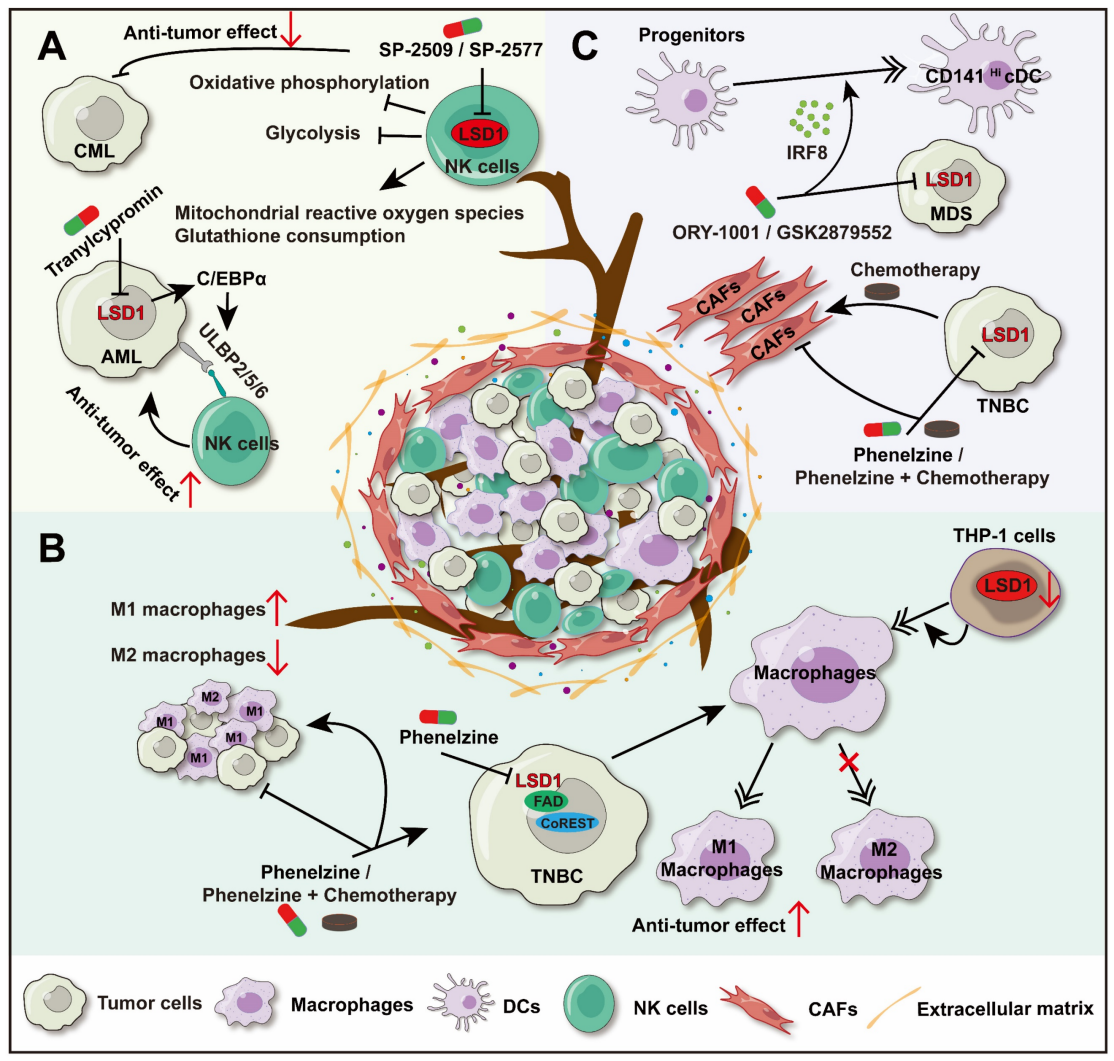
** LSD1's regulation on innate immunity**. A. LSD1 regulates the anti-tumor activity of NK cells through two pathways: LSD1 inhibition up-regulated the expression of NK cell ligand on AML; Scaffolding inhibitors reduce oxidative phosphorylation and glycolysis of NK cells and induce mitochondrial depletion of reactive oxygen species and antioxidant glutathione. B. LSD1 inhibition promotes the differentiation of TAM into M1 macrophages, promotes the infiltration of M1 macrophages, and inhibits the progression of TBNC. C. LSD1 inhibition decreased the proportion of CAFs in TME and inhibited the progression of TNBC. Inhibition of LSD1 can restore the expression of IRF8 in MDS progenitor cells, promote CD141^Hi^ cDCs differentiation.

NK cells are crucial innate lymphocytes involved in anti-tumor activity, antiviral defense, and immune regulation. They also participate in hypersensitivity reactions and autoimmune diseases. NK cells recognize and kill target cells, and activation receptors on their surface, such as NKG2D, NKP46, NKP30, and NKP44, play a key role in promoting their anti-tumor activity 68-70. Studies have found that LSD1 inhibitors can enhance the anti-tumor activity of NK cells by affecting their metabolism or the expression of NK cell ligands on tumor cells. This section will explore these mechanisms in detail (Figure 1A).

UL16-binding proteins (ULBPs), ligands of NKG2D, are expressed on the surface of tumor cells and can sensitize NK cells to kill these cells. The LSD1 inhibitor (Tranylcypromine) can upregulate the expression of ULBP2/5/6 by promoting CCAAT/enhancer-binding protein α (C/EBPα) expression in acute myeloid leukemia (AML) cells, thereby increasing NK cells ability to lyse AML cells 71. Similarly, the LSD1 inhibitor (GSK-LSD1) also enhanced the cytotoxicity and tumor infiltration of NK cells in diffuse intrinsic pontine glioma (DIPG) cells 72. However, there are variations in the effects of LSD1 inhibitors on NK cells activity. Studies have shown that scaffolding inhibitors (SP-2509 and SP-2577) can impair the ability of NK cells to lyse chronic myelogenous leukemia (CML). SP-2509 and SP-2577 reduce the oxidative phosphorylation and glycolysis of NK cells, induce the consumption of mitochondrial reactive oxygen species and the antioxidant glutathione, which lead to dissolution function of NK cells weakened. Moreover, scaffold inhibitors have certain toxicity to NK cells 72, 73.

These current study of LSD1's regulation on NK cells were based on LSD1 inhibitor, while the regulations of different inhibitors are not consistent. On one hand, inhibiting LSD1 (Tranylcypromine) promotes the infiltration of NK cells and enhances their cytotoxicity. On the other hand, scaffold LSD1 inhibitors can weaken the anti-tumor ability of NK cells and produce a certain toxicity to NK cells, leading to the development of resistance to immunotherapy in tumors. Further research is needed to explain the differences among LSD1 inhibitors. There is still much research space for the deep molecular mechanism of LSD1 on NK immunity.

### 3.2 Regulation of macrophages

Tumor-associated macrophages (TAM) are the most abundant infiltrating inflammatory cells in TME and play a key role in regulating the TME 74. TAM can differentiate into two phenotypes: M1 macrophages (tumor suppressor subtype) and M2 macrophages (tumor promoting subtype). While, the former exerting antitumor effects through cytotoxic activity and antigen presentation, and the latter can promote tissue repair, tumor cell proliferation, and inhibit anti-tumor immunity 75. Immunosuppressive cells in the TME are key contributors to tumor resistance against immune checkpoint blockade (ICB) therapy. They inhibit CD8+ T cell function and promote tumor growth 76. Targeting the highly infiltrated M2 macrophages could help overcome this resistance and enhance the anti-tumor effects in coordination with adaptive immunity.

LSD1 inhibitor (phenelzine), which targets both the flavin adenine dinucleotide (FAD) and CoREST binding domains of LSD1. It facilitates macrophages differentiate into M1 phenotype with antitumor effects in triple-negative breast cancer (TNBC) 77. In addition, phenelzine alone or combined with chemotherapy can increase the infiltration of M1 macrophages in the TME, while reduce M2 macrophages infiltration, and inhibit tumor growth consequently 78. In another study, LSD1 inhibition in THP-1 cells produced effects similar to those seen with the inhibitor. Downregulation of LSD1 induced the differentiation of THP-1 monocytes into macrophages by upregulating the methylation of the interleukin-6 (IL-6) promoter at H3K4. Further research on the impact of LSD1 on macrophage polarization is highly anticipated 79. Besides, interleukin-4 (IL-4) is a key cytokine creating an inflammatory inhibitory microenvironment in TAMs, inducing polarization of M2 macrophages 9. A study reveals that LSD1 was a co-activator of IL-4, and LSD1 inhibition reduces the expression of IL-4-induced genes in macrophages, but further research is needed to explore this phenomenon in TAMs 80 (Figure 1B).

LSD1 has been shown to play a key role in macrophage maturation 81, morphology maintenance 82, and inflammatory behavior across various disease conditions 83. While most current studies focus on LSD1's regulation of macrophage polarization, this opens new avenues for exploring how LSD1 influences macrophages in cancer. Given the high infiltration of macrophages in solid tumors, targeting LSD1 to enhance their anti-tumor activity presents a promising strategy for cancer immunotherapy.

### 3.3 LSD1 regulates innate immunity via DCs and cancer-associated fibroblasts (CAFs)

In solid tumors, CD141^Hi^ conventional dendritic cells (CD141^Hi^ cDCs) play a significant role in anti-tumor immune surveillance and immunotherapy response. In myelodysplastic syndromes (MDS), the level of CD141^Hi^ cDCs was positively correlated with the longer overall survival rate of MDS patients. Further studies found that there were fewer progenitors dedicated to DCs differentiation in MDS bone marrow, and these progenitors expressed lower levels of interferon regulatory factor-8 (IRF8), which is the primary regulator of CD141^Hi^ cDCs differentiation. However, inhibition of LSD1 (ORY-1001 or GSK2879552) can restore the expression of IRF8 in MDS progenitor cells and promote the differentiation of CD141^Hi^ cDCs 84 (Figure 1C). These data suggest that LSD1 has a significant role in modulating DCs.

Except for immune cells, LSD1 regulates the function of non-immune components in the TME. CAFs, a key part of the tumor microenvironment, are involved in angiogenesis, extracellular matrix remodeling, and immunosuppression, creating a supportive environment for tumor cells 85. Thus, targeting CAFs presents a novel approach to tumor therapy. Since CAFs are involved throughout the tumor immune process, they are tentatively grouped under the innate immunity segment in this review. Studies have shown that in breast cancer *in situ*, paclitaxel monotherapy increases the proportion of CAFs in the TME, while inhibition of LSD1 (Phenelzine) alone or combined chemotherapy reduces CAFs infiltration significantly 78. Meanwhile, abnormally elevated LSD1 expression in CAFs has been linked to poor patient survival 86, and direct inhibition of CAFs has been shown to reduce their activity and diminish their immunosuppressive function 87 (Figure 1C). All indications are that LSD1 may be an important cause of high infiltration of CAFs in the TME, resulting in immune escape.

Currently, the investigation into LSD1 within DCs and CAFs remains in its nascent stages. Limited studies have proposed that the inhibition of LSD1 could potentially facilitate the differentiation of DCs towards a more immunoreactive state and attenuate the tumor-supporting function of CAFs consequently exerting a pivotal role in innate immunity. Furthermore, this substantiates the notion that LSD1 serves as a promising target within the realm of innate immunity.

Based on the previous discussion, it is evident that LSD1 intersects with various aspects of the innate immune response. Thus, directing therapeutic interventions towards LSD1 may potentiate the innate immune system attributes of promptness, and affect inflammation within the context of tumor immunity, which vigilantly monitoring and eradicating the migration of tumor cells. What's more, the innate immune system can cascade towards activating, regulating, and imprinting memory responses in adaptive immunity through pathogen recognition and antigen representation and presentation. Hence, the potential utility of LSD1 inhibitors has come to the fore, as they hold promise in rendering tumor cells more readily discernible to the immune system, consequently augmenting the efficacy of immunotherapeutic agents such as immune checkpoint inhibitors.

## 4. LSD1 regulates adaptive immune processes

In a previous section, the role of LSD1 in regulating innate immune cells within the TME was emphasized. Moreover, it is widely acknowledged that effective anti-tumor defenses hinge on the activation of adaptive immune responses, which primarily orchestrated by T and B cells. T-cell-mediated immune response is a stepwise process involving multiple stages and coordinated mechanisms 88. (a) Antigen recognition and presentation: the antigen was released by cancer cell, and then recognized and presented to T cells by DCs or antigen-presenting cells (APCs). (b) T cell activation, proliferation, differentiation, and depletion: T cells begin to proliferate and differentiate into different T cell subtypes once receive signals from APCs. (c) T cell transport, invasion, effect stage: T cells transport and infiltrate into the tumor and stroma to recognize and kill cancer cells. However, any anomaly in any stage of this process can become a bottleneck in oncotherapy. The immunomodulatory role of LSD1 in these pathways has attracted much attention. Due to the limited reports on LSD1's regulation of B-cell-mediated immune responses, most studies have focused on its role in B-cell malignancies. Therefore, only a brief overview is provided in this paper. This chapter emphasizes the regulation of T-cell-mediated immune responses by LSD1 in cancer cells.

### 4.1 Regulation of T cells

#### 4.1.1 LSD1 affects antigen presentation and T cell activation

**Figure 2:**
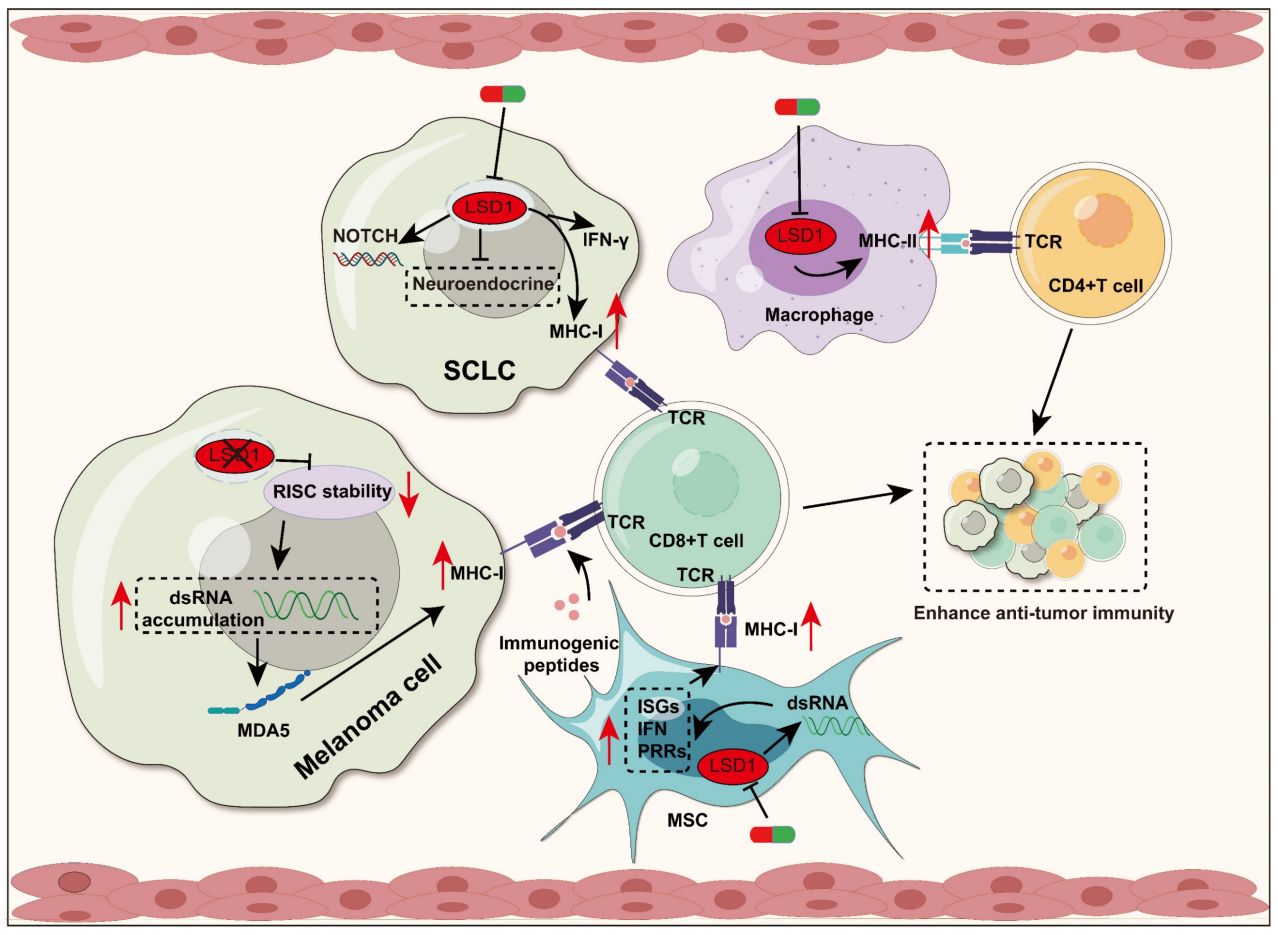
** LSD1 regulates antigen presentation and T cell activation.** Inhibition of LSD1 leads to upregulation of MHC-I expression in different cell types, promoting T cell activation and infiltration. In melanoma, loss of LSD1 results in upregulation of MHC-I gene expression. LSD1 deficiency leads to accumulation of dsRNA by reducing RISC components, which activate the IFN pathway subsequently, and then upregulate MHC-I expression. In SCLC, inhibition of LSD1 promotes enhanced tumor immunogenicity and increased CD8+ T cell activation and infiltration, which is achieved by restoring MHC-I expression, triggering intrinsic IFN signaling, and other pathways. In MSCs, LSD1 inhibition also promotes MHC-I expression by inducing dsRNA stress, while upregulating the level of ISGs, IFN, and PRRs. Additionally, Inhibition of LSD1 up-regulates the expression of MHC-II in macrophages and promotes antigen presentation to CD4+T cells.

Antigen presenting process is the key to T cell activation. The antigens were presented by the MHC, and recognized by T cells through specific T cell receptor (TCR), causing T cell activation. MHC-I mainly presents antigens to CD8+ T cells, while MHC-II mainly presents antigens to CD4+ T cells 89, 90. In many cancer types, tumor cells can diminish or completely abolish the expression of MHC-I molecules through diverse mechanisms. Consequently, this renders tumor cells incapable of being effectively identified and targeted by CD8+ T cells, leading to immune evasion, namely, escaping immune surveillance and attack by the host. Enhancing the antigen presentation process was a crucial step in the anti-tumor immunotherapy 91, 92. Studies have shown that LSD1 can affect the antigen presentation process by regulating the expression of MHC-I and MHC-II molecules, and this will be discussed in the following sections (Figure 2).

In melanoma cells, LSD1 deletion enhances T cell activation and infiltration by upregulating the expression of MHC-I encoding genes. Mechanistically, LSD1 deficiency reduces the activity of RNA-induced silencing complex (RISC) components, leading to the accumulation of double-stranded RNA (dsRNA). This dsRNA stress activates MDA5, which subsequently upregulates MHC-I and triggers type 1 interferon (IFN) responses 34. Small cell lung cancer (SCLC) is a highly invasive neuroendocrine tumor characterized by early acquired treatment resistance and limited benefits from ICB. The inhibition of MHC-I is a key mechanism for T cell immune therapy resistance in SCLC 93. Inhibition of LSD1 has been shown to promote the conversion from a high neuroendocrine (NE) gene expression to a low NE phenotype in SCLC, improving NOTCH signaling activation. Additionally, LSD1 inhibition significantly restored MHC-I expression and triggered intrinsic IFN signaling, both of which enhanced tumor immunogenicity and significantly increased CD8+ T cell activation and infiltration 36, 37. In addition, LSD1 inhibition also up-regulates MHC-I class molecule-mediated antigen presentation in mouse breast tumor cells. However, the exact mechanism is not reported in detail 94.

The regulation of MHC-I and MHC-II molecules by LSD1 is not only limited in tumor cells, but also in mesenchymal stem cells (MSCs). It has been reported that inhibition of LSD1 can result in the upregulation of dsRNA stress and its related response elements in MSCs, which include pattern recognition receptors (PRRs), Type I IFN, and IFN-stimulating genes (ISGs). Depending on these, LSD1 inhibition can enhance the ability of MSCs to present immunogenic peptides to CD8+T cells by inducing the expression of MHC-I molecules on the surface of MSCs, which induces an effective anti-tumor immune response in consequence 95. In addition, LSD1 inhibition can upregulate macrophage CD80/86, MHC-II genes 77. Further research on whether targeting LSD1 can enhance macrophage antigen presentation to CD4+ T cells and boost the anti-tumor immune response is highly anticipated 96.

In short, elevated LSD1 expression can suppress the antigen-presenting process of tumor cells, thereby attenuating the recognition and cytotoxicity of T cells against tumor. Consequently, utilizing LSD1 inhibitors can enhance tumor immunogenicity and thereby promote anti-tumor immunity.

#### 4.1.2 LSD1 affects the differentiation, infiltration and depletion of T cells

As previously discussed, LSD1 plays a critical role in governing the activation, differentiation, infiltration, and depletion of T cells within the context of anti-tumor immunity. There is literature suggesting that inhibiting LSD1 can induce the differentiation of CD4+ T cells into Th1 cells by upregulating Runx2, Tbx21, and Eomes in CD4+T cells. This process may have a promoting effect on tumor immunity 97. Additionally, targeting LSD1 can enhance CD8+ T cell infiltration into tumors and boost the anti-tumor immune response through various pathways, such as increasing chemokine expression 98-100, downregulating PD-L1 33, and accumulating dsRNA 34, 95. Particularly excitingly, precise and transient LSD1 inhibition during T cell receptor activation and Interleukin-2 (IL-2) signaling significantly enhanced the memory phenotype of mouse CD8+ T cells. This intervention also enabled these cells to produce multiple cytokines, resist depletion, and sustain antigen-dependent and independent persistence post adoptive transfer 101. Meanwhile, research has shown that targeting LSD1 induces extensive epigenomic remodeling in T cells by modulating their transcriptional profiles, which promotes T cell phenotypic stability and function 102. In summary, altering the status of LSD1 can serve as a potential anti-cancer strategy. This strategy enhances T cell activation, differentiation, and infiltration through multiple mechanisms, reverses T cell depletion, and thus enhances T cell attack on tumors. To better understand the role of LSD1 in tumor immunity, the regulatory effects of LSD1 on T cells are summarized in Table 1.

### 4.2 Regulation of B cells

While the crucial role of T cells in tumor immunity is well understood, the contribution of B cells is often overlooked. In fact, B cells have significant prognostic value and have become an important predictor of immune checkpoint inhibitor response in a variety of cancers. B cells have a variety of functions, such as antigen presentation, antibody production, regulation of the TME, and promote immune cells to attack tumor cells 109. LSD1 has been reported to be necessary for the proliferation and differentiation of B cells 110, 111. Interestingly, the role of LSD1 in regulating B cell differentiation was not always coincident. On the one hand, in germinal center-derived lymphoma, conditional loss of LSD1 can inhibit the constitutive expression of BCL6 and promote the expression of genes related to proliferation of germinal center B cells, which further inhibit the differentiation of germinal center B cells and delay the development of BCL6-driven lymphoma significantly 112. In addition, inhibition of LSD1 has been shown to improve proteasome inhibitor sensitivity and overcome drug resistance in patients with multiple myeloma (MM). Therefore, LSD1 inhibitor combined with proteasome inhibitor may have the potential to be expansibility used to other B cell malignancies treatment 113. Meanwhile, it was also reported that LSD1 could inhibit disease progression by regulating abnormal plasma cells, which were effector B cells 114. Currently, LSD1's regulation of B cells is mainly studied in the context of B-cell malignancies. We review this chapter and suggest that in the future more attention should be paid to the potential role of LSD1 in the regulation of tumor-killing B cells, which would be desirable or be used for reference or guidance.

## 5. LSD1 plays a crucial role in tumor immunotherapy

Immunotherapy has advanced rapidly in recent years, offering tremendous potential to combat tumors by modulating the human immune system. 115. The main methods include: (a) Immunomodulators, which regulate cell-cell interactions of the immune system, tumor immunosurveillance and clearance processes, such as chemokines and cytokines 116, 117; (b) ICB therapy, which activates the immune system to attack tumor cells by inhibiting checkpoint molecules, such as CTLA-4, PD-L1, and CD47 118; (c) Cell therapy, a type of cell therapy in which live cells are injected into the patients' body to enhance the immune effect to treat the disease, such as T-cells, NK-cells, and stem cells 119; (d) Cancer vaccines, which prepare tumor-specific antigens to stimulate the immune system to produce an immune response against the tumor 120.

It was mentioned in the previous chapter that LSD1 can regulate immune cell differentiation and function, and weaken immune response in some tumors. Inhibition of LSD1 activity can enhance the function of immune cells and improve the efficacy of immunotherapy, making LSD1 a potential immune regulatory target. This chapter will primarily review the effects of LSD1 on immunomodulators and immune checkpoint molecules, as well as the efficacy of combining LSD1 inhibitors with ICB therapy or CAR-T therapy, based on existing studies.

### 5.1 Regulation of immunomodulators

Tumor-associated immune factors are biologically active molecules related to tumor occurrence, development, and immune regulation. They play essential roles in processes such as tumor immune surveillance and immune escape. For instance, cytokines (such as IL-6, IL-10, transforming growth factor-β (TGF-β), vascular endothelial growth factor (VEGF) and chemokines (such as CXCL9, CXCL10) are represented, and these factors can regulate the effective function of T cells, thereby changing the balance of tumor immune response 121-126.

Highly expressed TGF-β has immunosuppressive effects, promoting tumor cells to evade immune surveillance 127. Meanwhile, TGF-β can also enhance tumor vascular endothelial cell proliferation and angiogenesis, creating a favorable microenvironment by providing oxygen and nutrition, which further promote tumor development 128, 129. Inhibiting TGF-β signaling can interrupt the epithelial-mesenchymal transition (EMT) process, enhance tumor immune response, and reduce the risk of tumor metastasis, which has broad clinical application prospects 130-132. LSD1 has been reported to have a significant effect on TGF-β. In GC, reducing LSD1 can decrease the expression of TGF-β 63. In NSCLC, LSD1-mediated demethylation of SEPT6 protein promotes cancer cell metastasis by activating the TGF-β1 pathway 62. However, the positive correlation between LSD1 and TGF-β in GC and NSCLC has not been deeply studied in immune regulation. Conversely, LSD1 and TGF-β show a negative correlation in breast cancer. Similarly, it is also not clear whether LSD1 regulates immunity accordingly 133. Meanwhile, the absence of LSD1 promotes TGF-β expression in melanoma and colorectal cancer (CRC) cells, and the up-regulated TGF-β counteracts the anti-tumor effects of LSD1 deletion-induced T cell infiltration by inhibiting the cytotoxicity of CD8+ T cells within the tumor. Simultaneous elimination of LSD1 and TGF-β, combining with PD-1 blockade, can significantly enhance CD8+ T cell infiltration and cytotoxicity 61.

VEGF is a key survival factor and mitogen for vascular endothelial cells. In the VEGF family, VEGF-A and VEGF-C play important roles in tumor immunity. There is extensive research on VEGF-A, and the term "VEGF" is often referred to VEGF-A 134. However, VEGF-A possesses certain immunosuppressive properties, as its upregulation can promote T cell exhaustion, inhibit DCs maturation, and enhance the recruitment of regulatory T (Treg) cells and tumor-promoting M2 macrophages 135. In contrast, VEGF-C often exhibits positive effects in tumor immunity, such as promoting the activation of CD8+T cells in brain tumors. Furthermore, it enhances the chemotherapeutic efficacy and anti-tumor immunity against brain tumors significantly 136. LSD1 actively regulates VEGF expression in various cancers. In GC, downregulating LSD1 can inhibit the metastatic potential of GC cells and activate the VEGF-C-mediated PI3K/AKT signaling pathway 137. In prostate cancer (PC), LSD1 upregulation is associated with PC recurrence and VEGF-A upregulation 138. Additionally, in human breast cancer, stable HIF-1α induced by LSD1 interacts synergistically with CBP and MTA1 to promote tumor angiogenesis induced by VEGF, providing continuous oxygen and nutrition for tumor growth 139. However, according to current research, it remains unclear whether LSD1 regulating VEGF leads to immunosuppression.

Chemokines are a class of cytokines that regulate immune cell migration and lymphoid tissue development 140, 141. In the development of cancer, they play a core role in guiding immune cells infiltrate to tumor sites, shaping the immune characteristics of the TME, and often inhibiting tumor growth. Additionally, chemokines can also directly act on non-immune cells in the TME, such as tumor cells, stromal cells, and vascular endothelial cells 142, 143. In TNBC, inhibiting LSD1 enhances the enrichment of H3K4me2 in the promoter regions of chemokines CCL5, CXCL9, and CXCL10, which promotes the attraction of CD8+ T cells into the TME and exerts strong killing power 99. This result was further confirmed in a recent study 98. In small cell carcinoma of the ovary hypercalcemic type (SCCOHT), inhibiting LSD1 enhances the release of cytokines IFN-γ, IFN-β, IL12p70, IL-1β, IP-10 (CXCL10), IL-10, IL-2, IL-6, MCP-1 (CCL2), and IL-8 (CXCL8), revealing the important role of LSD1 inhibition in promoting antitumor immunity 144.

**Figure 3:**
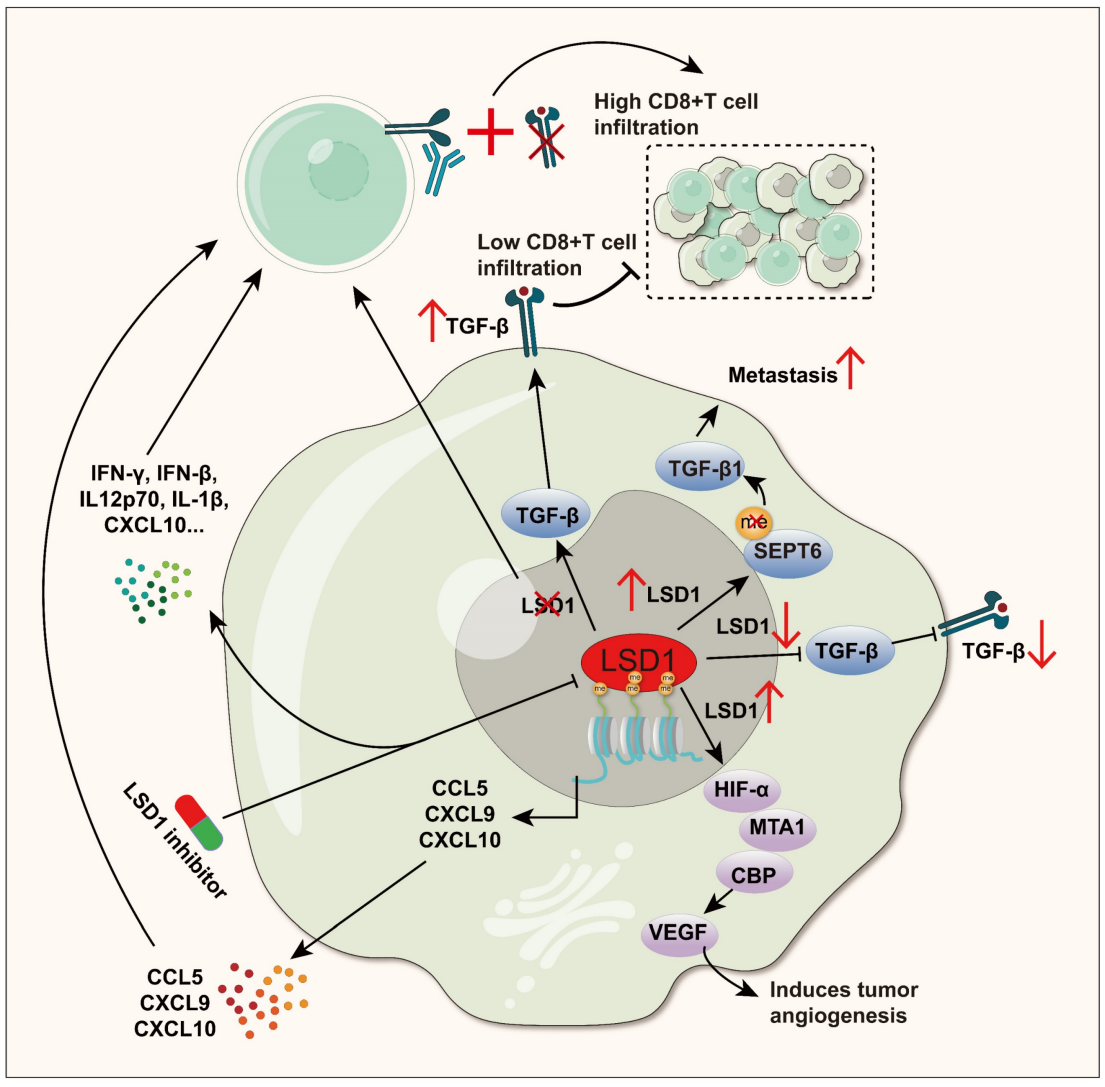
** LSD1 interferes with the anti-tumor immune process by regulating the expression of TGF-β, VEGF and Chemokines.** TGF-β: In GC and NSCLC, LSD1 positively regulates TGF-β and promotes cancer cell metastasis, while in melanoma and CRC, LSD1 negatively regulates TGF-β, while eliminating LSD1 and TGF-β and jointly blocking PD-1 can significantly enhance CD8+ T cell infiltration and cytotoxicity. VEGF: Stabilized by LSD1, HIF1α collaborates with CBP and MTA1 to boost VEGF-triggered angiogenesis in human breast cancer. Chemokines: In TNBC and SCCOHT, inhibition of LSD1 can enhance the secretion of chemokines such as CCL5, CXCL9, CXCL10 and CCL2, and recruit CD8+ T cells into the TME to play a powerful killing ability.

Overall, LSD1 promotes tumor progression by directly or indirectly influencing TGFβ, VEGF, and chemokines (Figure 3). Cytokines are key mediators of cellular communication in the TME, crucial for activating and regulating innate and adaptive immune responses 145. For example, as mentioned earlier, simultaneous inhibition of LSD1 and TGF-β, along with PD-1 blockade in melanoma and CRC, significantly enhanced CD8+ T cell infiltration and cytotoxicity. In TNBC, LSD1 inhibition also increased the expression of chemokines CCL5, CXCL9, and CXCL10, recruiting CD8+ T cells into the TME, further strengthening the adaptive immune response. Cytokines are crucial pathways through which LSD1 influences immune cells in the TME, yet they remain less studied. A promising new direction is to further explore how LSD1 modulates cytokines to influence tumor immunity. This, combined with current insights into LSD1's effects on innate and adaptive immunity, could lead to new therapeutic strategies to enhance anti-tumor immunity.

### 5.2 LSD1 and ICB therapy

#### 5.2.1 Regulation of immune checkpoint molecules

Immune checkpoint molecules, including PD-1, PD-L1/PD-L2, CTLA-4, and CD47, serve as negative co-stimulatory factors regulating the duration of immune responses. Dysregulated expression of these molecules can disrupt normal immune function 146. Current studies on LSD1's impact on immune checkpoints predominantly center around PD-1/PD-L1, with varying regulatory dynamics observed across different cancers, indicating intricate and diverse regulatory mechanisms. In GC, LSD1 exhibits positive regulation of PD-L1, whereas in melanoma, the relationship is reversed 33, 34. Furthermore, the association between LSD1 and other immune checkpoint molecules like CTLA-4 remains unclear. Additionally, the synergistic effects of LSD1 inhibitors in combination with ICB, notably anti-PD-1/PD-L1 therapies, have been extensively documented for their significant efficacy across various cancer types. The expression of PD-1, PD-L1 and other immune checkpoint molecules can directly affect the efficacy of ICB, and numerous studies have highlighted LSD1's pivotal role in modulating the expression of these molecules. Thus, Table 2 meticulously outlines LSD1's biological impacts on tumor immunity and immunotherapy by detailing its influence on immune checkpoint molecule expression in various cancers.

#### 5.2.2 LSD1 inhibitors combined with ICB therapy

ICB, as one of the most successful immunotherapies, has been approved for several oncology indications. However, its overall efficacy is not ideal, with only 20% of patients with solid tumors achieved complete remission after treatment 151. The expression of immune checkpoint molecules on tumor cells is closely related to the clinical response of ICBs 152. As previously mentioned, inhibition of LSD1 regulates the expression of immune checkpoint molecules in immune cells as well as tumor cells, suggesting that LSD1 inhibitors show promising potential as a combinatory treatment in ICB therapy.

**Figure 4:**
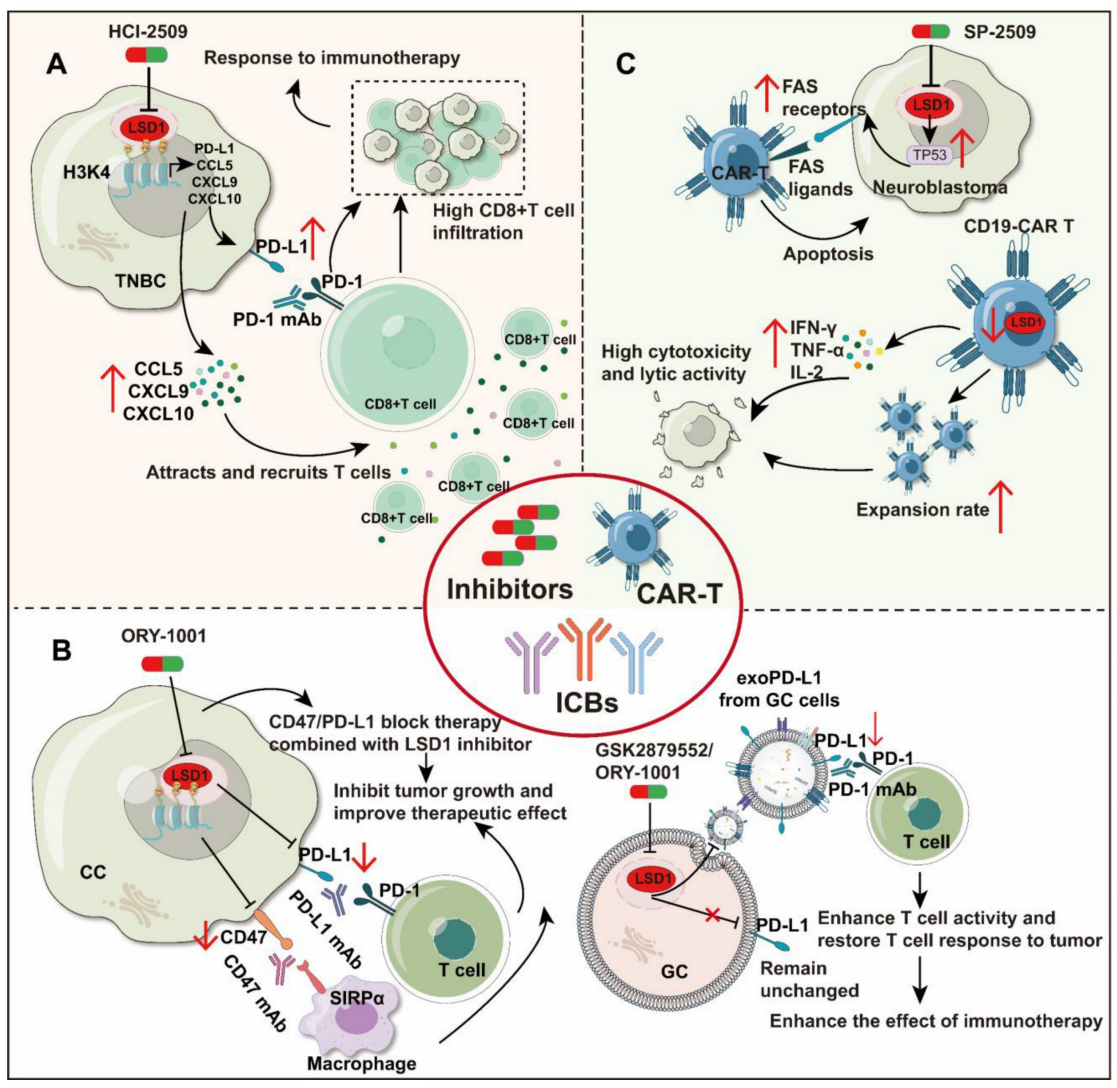
** LSD1 plays a crucial role in tumor immunotherapy.** A. In TNBC, inhibition of LSD1 resulted in increased expression of CCL5, CXCL9, CXCL10, and PD-L1, which attracted CD8+ T-cells into the TME and acted as killers. The combination of HCI-2509 and PD-1 antibodies further enhanced the therapeutic effect. B. Inhibition of LSD1 results in decreased expression of PD-L1 and CD47 on the surface of CC tumor cells. The therapeutic effect of LSD1 inhibitors is enhanced when used in conjunction with PD-L1 or CD47 antibodies. Lack of LSD1 results in decreased expression of PD-L1 in GC exosomes, increased T cell activity, and restoration of T cell ability to attack tumors. C. Inhibition of LSD1 in tumor cells enhanced CAR T cell cytotoxicity against neuroblastoma, whereas LSD1 inhibition in CD19-CAR T cells also promoted their proliferation rate and cytokine secretion.

As an illustration, in TNBC (Figure 4A), inhibition of LSD1 (HCI-2509) resulted in elevated expression of T cell chemokines (CCL5, CXCL9, CXCL10) and PD-L1. These chemokines facilitated infiltration of CD8+ T cells into the TME, where they exerted cytotoxic effects. Moreover, compared to PD-1 antibodies alone, combining HCI-2509 with PD-1 antibodies markedly increased CD8+ T cell infiltration and efficacy in overcoming PD-1 antibodies resistance 99. Conversely, in CC (Figure 4B), inhibition of LSD1 (ORY-1001) directly reduced PD-L1 and CD47 on the surface of tumor cells. Inhibiting LSD1 in combination with either anti-CD47 or PD-L1 treatments was more effective in inhibiting tumor growth compared to using a single blockade strategy 35.

In addition, exosomal PD-L1 plays a pivotal role in ICB therapy. It inhibits cytokine production and promotes T-cell apoptosis, enabling tumors to evade immune system attacks. Therefore, reducing exosomal PD-L1 levels may enhance patient sensitivity to PD-L1/PD-1 therapy and enhance immune efficacy 153, 154. In GC (Figure 4B), deletion of LSD1 reduces PD-L1 expression in exosomes but does not affect PD-L1 on the cell membrane of gastric cancer cells. Inhibition of LSD1 (GSK2879552 and ORY1001) prevents the translocation of exosomal PD-L1, improves T-cell activity, and restores T-cells to attack tumors, overcoming the immunosuppression 33. Furthermore, the deletion of RNF20 was observed to render cancer cells more sensitive to PD-1 antibody in mouse breast cancer. However, ectopic expression of LSD1 was demonstrated to be capable of reversing this phenomenon 155. This indicates that the combination of LSD1 inhibitors with PD-1 antibodies may hold therapeutic promise. Throughout the above report, an intriguing phenomenon has emerged: regardless of the regulatory relationship between LSD1 and PD-L1, the combined blockade of PD-(L)1 and inhibition of LSD1 has shown effectiveness in suppressing tumor growth across various cancer types. Table 3 provides a comprehensive overview of LSD1 inhibitors pivotal role in enhancing tumor immunotherapy. In conclusion, LSD1 inhibition has emerged as a potent adjunctive approach in immunotherapy, facilitating the conversion of "cold tumors" to "hot tumors" and mitigating immunotherapy resistance.

#### 5.2.3 LSD1 inhibitors combined with CAR-T therapy

CAR-T cell therapy has made important breakthroughs in the field of immunotherapy, mainly applied to hematological malignancies such as lymphoma and leukemia 156. However, scaling up CAR-T therapy to solid tumors remains a challenge. The efficacy of CAR-T therapy in solid tumors is limited by a variety of factors, such as antigen heterogeneity and loss, limited potency and persistence, poor infiltration ability, and TME inhibition 157. Combining small molecule inhibitors with CAR-T cells treatment may be a promising new therapeutic strategy in solid tumors, and LSD1 is a good option for CAR-T cell combination therapy.

Studies have confirmed that inhibition or deletion of LSD1 can enhance the lethality of CAR-T cells to tumor cells 158, 159 (Figure 4C). In neuroblastoma, inhibiting LSD1 (SP-2509) in tumor cells can increase the expression of FAS receptors on the tumor cell surface by promoting TP53 mediated gene transcription activation. Therefore, FAS ligands on CAR-T cells can bind to FAS receptors on the tumor cell surface even without antigen expression, attacking tumor cells consequently 158. Moreover, priming with LSD1 inhibitors (GSK-LSD1) increases the persistence and antitumor efficacy of human CD19-CAR T cells in both leukemia and melanoma models 101. Meanwhile, in lymphoma, knocking down LSD1 in CD19-CAR T cells can also significantly improve the anti-tumor effect by maintaining the proliferation rate of CD19-CAR T cells, then promote their secretion of IFN-γ, tumor necrosis factor-α (TNF-α) and IL-2, and enhance cytotoxicity and lytic activity 159. Thus, pharmacological inhibition of LSD1 could be exploited to improve adoptive T cell therapy (Table 3).

These studies show that the anti-tumor effect of CAR T cells can be improved by inhibiting LSD1 activity, which providing a new auxiliary strategy for improving the efficacy of CAR T cell therapy, and giving a new idea for CAR T cell design. However, the above findings need further clinical verification to determine the feasibility and safety of clinical application.

## 6. Conclusion and perspective

In this review, the applications of LSD1 in remodeling tumor immunity and its significant potential in tumor immunotherapy are comprehensively summarized. LSD1 has the unique ability to regulate both the innate and adaptive immune systems. An in-depth analysis of the literature indicates that LSD1 is a promising target for tumor immunoregulation and immunotherapy.

Currently, the significance of LSD1 inhibition in cancer therapy lies primarily in its multifaceted impact on the immune system. Here are the key points: (a) Innate immune effects: LSD1 regulates macrophage polarization and promotes their differentiation towards inflammatory phenotypes. Enhanced NK cell activity: Inhibiting LSD1 is anticipated to augment NK cell cytotoxicity against tumor cells, albeit the precise molecular mechanisms necessitate further investigation. (b) Adaptive immune regulation: LSD1 is pivotal in T cell-mediated immune processes. Specifically, LSD1 inhibitors can reinstate the expression of MHC-I molecules on tumor cell surfaces, thereby aiding in the activation and enhancement of T cell immune responses against tumors. (c) Immune checkpoint regulation: Inhibition of LSD1 can regulate the expression of PD-L1 on the surface of tumor cells, which is crucial for enhancing the efficacy of anti-PD-1/PD-L1 immunotherapy. Specifically, inhibiting LSD1 may mitigate tumor immune escape to T cells, making immune checkpoint inhibitor therapy more effective. In conclusion, targeted therapy against LSD1 holds significant promise in augmenting tumor immunogenicity, boosting immune cell activity, and optimizing immune checkpoint efficacy, thereby positioning it as a promising candidate for future synergistic immunotherapy strategies.

However, the research on targeting LSD1 in anti-tumor immunity still has a long way to go. For instance, various classes of LSD1 inhibitors exhibit opposite impacts on NK immunity. Scaffold LSD1 inhibitors (reversible) can induce toxicity in NK cells and impair the ability of NK cells to lysate tumor cells. Conversely, the irreversible LSD1 inhibitor can effectively enhance the lytic effect of NK cells on tumor cells, while with minimal toxicity to NK cells. This may be that reversible LSD1 inhibitors impair the metabolic function of NK cells. Hence, future studies on LSD1 regulation of NK cells should focus on the detrimental effects of reversible LSD1 inhibitors. Furthermore, in numerous human malignancies, T cell presence within tumor lesions correlates with improved patient prognosis. However, T cell infiltration is often poor in solid tumors, leading to suboptimal responses to T cell immunotherapy 161.

In the process of reviewing the literature, we have found that targeting LSD1 in combination with anti-PD-1/PD-L1 therapy not only enhances treatment efficacy but also promotes T cell infiltration and activation in the TME. Further studies have highlighted the controversial role of LSD1 in regulating the PD-1/PD-L1 axis across different cancer types. This may be linked to the complex role of LSD1 in the TME. PD-L1 expression is regulated by various signaling pathways, and LSD1 can influence its levels by targeting key nodes within these pathways. Additionally, different cancers have distinct TME and signaling networks, which may contribute to the variability in LSD1's effects. Thus, the potential of combining LSD1 inhibitors with ICBs in various cancer types warrants further discussion. In cancers where LSD1 and PD-L1 are positively feedback-regulated, such as GC and HCC, LSD1 inhibitors can reduce tumor immune escape by downregulating PD-L1 expression. When used in combination with ICBs, this may enhance T-cell activity and amplify the anti-tumor immune response by weakening the immunosuppressive TME. Conversely, in cancers where LSD1 and PD-L1 are negatively feedback-regulated, such as melanoma and NSCLC, high LSD1 expression inhibits PD-L1 antigen presentation, which results in a low immunotherapy response. In these cases, LSD1 inhibitors may restore the sensitivity and response to ICB therapy. This dual action could make combination therapy more effective in certain immunosuppressive TME. In addition, to this, LSD1 inhibitors may also indirectly affect the PD-1/PD-L1 signaling pathway by modulating other immune-related pathways, such as NK cell activity or macrophage polarization, but this is only one of our hypotheses. In this context, LSD1 inhibitors may synergize with ICBs to remodel the TME at multiple levels, thereby enhancing therapeutic efficacy. Moreover, macrophages play a crucial role in maintaining tissue homeostasis and the TME, exhibiting the highest infiltration rate among immune cells in solid tumors 162. Research indicates that LSD1 inhibition promotes the inflammatory polarization of macrophages, potentially enhancing T cell infiltration and cytotoxicity. Consequently, combining LSD1 targeting with T cell immunotherapy holds promise for enhancing immunotherapy efficacy in solid tumors via highly infiltrated macrophages. Finally, despite the challenges posed by solid tumors to the expansion of CAR-T therapy applications, combining CAR-T therapy with immune checkpoint therapy has shown significant tumor inhibition in select cancer patients. However, variability in PD-1, PD-L1, and CTLA-4 expression levels limits the overall efficacy of CAR-T therapy combined with ICB therapy 163. Meanwhile, TGF-β as a critical immunosuppressive factor for T cells, several clinical trials that TGF-β targeting combined with CAR-T therapy have been approved (NCT00889954, NCT04227275, NCT03089203, NCT03198546) 164-167. Addressing this issue, LSD1 served as the upstream regulator of PD-L1 and TGF-β, and targeting LSD1 inhibition holds the potential to down-regulate both factors in certain cancer types. Therefore, it is anticipated and exciting to study whether the addition of LSD1 inhibitors to the dual-combination regimen that targeting TGF-β combined with CAR-T therapy will produce a better anti-tumor effect.

In general, this review systematically summarizes the role of LSD1 in tumor immunity from the perspective of innate immunity and adaptive immunity for the first time, highlighting LSD1's pivotal role as a bridge between these immune pathways. And this provides new perspectives and a theoretical foundation for exploring its potential applications in tumor immunotherapy.

## Figures and Tables

**Table 1:** LSD1 affects T-cell function

	Cancers	Molecular mechanism	Biological function	References
T cell activation and Infiltration	Melanoma	LSD1 deletion up-regulates MHC-Ⅰ expression	Promote the activation and infiltration of CD8+T cells	34
	SCLC	LSD1 inhibition enhances the induction effect of IFN-γ on MHC-I, and then increases the expression level of MHC-I	Enhance the activation and infiltration of CD8+ T cells	36
T cell activation	TNBC	LSD1 inhibition upregulates MHC-I expression	Inhibit breast cancer progression	94
	TNBC	Inhibition of LSD1 up-regulates macrophage MHC-II	Promotes antigen delivery to CD4+ T cells, thus promoting anti-tumor immunity	77
T cell differentiation	/	Inhibition of LSD1 increased the expression levels of Runx2, Tbx21, Eomes and H3K4 in CD4+ T cells	Promote the development of CD4+ T cells to Th1 cells secreting IFN-γ, and promote tumor immunity	97
T cell Infiltration	Melanoma and CRC	/	Simultaneous inhibition of LSD1 and TGF-β combined with anti-PD-1 therapy significantly increased the infiltration and cytotoxicity of CD8 + T cells	61
	NSCLC	Inhibition of LSD1 promotes ERGIC1-mediated IFNGR1 stabilization and membrane transport, leading to upregulation of MHC-I/PD-L1 in tumors	Inhibition of LSD1 combined with anti-PD-1 therapy promoted CD8+T cell infiltration and inhibited the progression of NSCLC	103
	TNBC	Inhibition of LSD1 up-regulates CCL5, CXCL9, CXCL10 and PD-L1	LSD1 inhibition combined with anti-PD-1 decreased Ki-67 levels in TNBC and promoted CD8+ T cell infiltration	99
	Melanoma	Inhibition of LSD1 increased the number of Slamf6+Tim-3-Texprog cells in tumors and increased the expression level of Tcf1 in ID2-deficient CD8+ T cells.	Inhibition of LSD1 can save the damage of CD8+ T cells caused by the deletion of Id2 gene, and the efficacy of anti-PD-1 therapy is low.	104
T cell depletion	Melanoma	Inhibition of LSD1 promotes the production of effector cytokines and decreases the expression of related inhibitory receptors.	Inhibition of LSD1 reverses T cell exhaustion and enhances T cell persistence and anti-tumor efficacy.	101
T cell killing ability	GC	/	Inhibition of LSD1 enhanced the sensitivity of T cells to GC cells, improved its killing ability to GC cells, and inhibited tumor progression	105-108

SCLC: Small cell lung cancer; NSCLC: Non-small cell lung cancer; TNBC: Triple negative breast cancer; GC: Gastric cancer; CRC: Colorectal cancer

**Table 2:** LSD1 affects the expression of immune checkpoints

Cancers	Regulation	Immune checkpoints	Function and mechanism	References
Melanoma	Negative	PD-1	1. LSD1 loss leads to an increase in PD-1-expressing CD8 T cells in tumor infiltration	147
CRC	Positive	PD-1	1. LSD1 deficiency enhanced the survival rate of CD8+ tumor-infiltrating leukocytes (TILs) progenitor cells and decreased the expression of PD-1 protein on the surface of CD8+ TILs cells, without affecting the frequency of PD-1+ cells	102
Melanoma	Negative	PD-L1	1. Knockout of LSD1 enhances the expression of PD-L1 in tumor cells, and combined therapy with anti-PD-1 can overcome tumor resistance to PD-1 blocking, thus playing a significant tumor inhibitory effect	34
TNBC	Negative	PD-L1	1. Inhibition of LSD1 leads to increased enrichment of H3K4me2 on chemokines and PD-L1 promoters, thereby up-regulating the expression of PD-L1 and promoting the release of t cell chemokines (CCL5, CXCL9, CXCL10)	99
HNSCC	Negative	PD-L1	1. LSD1 deletion induced PD-L1 expression in tumor cells and inhibited tumor stem-like features 2. Combined treatment with LSD1 inhibitor and anti-PD-1 can reduce Ki-67 level, promote CD8+T cell infiltration, overcome tumor immune evasion, and significantly inhibit tumor growth	100
OCCC/SCCOHT	Negative	PD-L1	1. Inhibition of LSD1 can enhance the expression of tumor PD-L1, while combined anti-PD-L1 treatment can significantly increase the distribution of CD8+T cells in tumor infiltrating, and enhance the anti-tumor effect	144
OSCC	Negative	PD-L1	1. Inhibition of LSD1 can enhance the expression of PD-L1 in tumors, and combined use of YAP inhibitors can further increase the expression of PD-L1 2. The combination of LSD1 inhibition and anti-PD-1 /PD-L1 treatment can inhibit tumor growth more effectively	148
SCLC	Negative	PD-L1	1. Inhibition of LSD1 activates NOTCH pathway, weakens neuroendocrine features of SCLC, and enhances SCLC's response to PD-1 therapy 2. Inhibition of LSD1 increased the expression of tumor PD-L1, and combined with anti-PD-L1 treatment significantly enhanced the infiltration and anti-tumor effect of CD8+ T cells	36, 37
CC	Positive	PD-L1	1. Decreased H3K4me2 levels in CD47 and CD274 promoters activated by LSD1, directly decreased the expression of CD47 and PD-L1 2. The combination of LSD1 inhibitor and anti-PD-L1 can significantly enhance the effect of inhibiting tumor growth	35
HCC	Positive	PD-L1	1. LSD1 interacts with myocyte enhancer Factor 2D (MEF2D) to reduce its methylation, causing demethylated MEF2D to bind to the promoter of PD-L1 and activate its expression 2. miR-329-3p targets LSD1 mRNA and reduces its expression to inhibit the expression of PD-L1, thereby enhancing the lethal effect of T cells on HCC cells	149
GC	Positive	PD-L1	1. Both inhibition of LSD1 and decrease of LSD1 expression could decrease the level of PD-L1 in GC cells 2. Loss of LSD1 inhibits tumor growth by reducing exosome PD-L1 and increasing T cell activity	33, 105-107
LUAD	Positive	PD-L1	1. Inhibition of LSD1 reduces PD-L1 expression through JAK pathway, thereby inhibiting the proliferation, migration and tumor growth of LUAD cells	150
CC	Positive	CD47	1. The decrease of LSD1 increases the H3K4me2 level in CD47 promoter, which directly leads to the decrease of CD47 expression 2. LSD1/ wild-type p53/miR-34a signal axis targets the 3' untranslated region (3' utr) of CD47, which is involved in regulating the expression of CD47 3. The combination of LSD1 inhibitor and anti-CD47 treatment is more effective in inhibiting tumor growth than the single use strategy	35
OCCC/SCCOHT	/	CTLA-4	1. The combination of LSD1 inhibitor and anti-CTLA-4 significantly improved PBMC permeability in SWI/SNF mutant ovarian cancer	144

SCLC: Small cell lung cancer; NSCLC: Non-small cell lung cancer; TNBC: Triple negative breast cancer; GC: Gastric cancer; CRC: Colorectal cancer; HNSCC: Head and Neck Squamous Cell Carcinoma; OCCC: Ovarian clear cell carcinoma; SCCOHT: Small cell carcinoma of the ovary hypercalcemic type; OSCC: Oral squamous cell carcinoma; HCC: Hepatocellular carcinoma; CC: Cervical cancer; LUAD: Lung adenocarcinoma

**Table 3:** Application of LSD1 inhibitors in the field of tumor immunity

Name	Structure	Type	Functions	References
GSK-LSD1	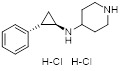	Irreversible LSD1 inhibitor	1. Promote CD8+ T cell infiltration and antitumor response 2. Stimulate antigen presentation and enhance T cell-mediated cytotoxicity 3. Enhanced intra-tumoral persistence of exhausted T cells 4. Enhance the persistence and antitumor efficacy of human CD19-CAR T cells.	34, 94, 101
ORY-1001 (Phase 1/2)	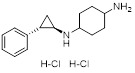	Irreversible LSD1 inhibitor	1. Increase the expression level of MHC-I on the surface of tumor cells, promoting the transcription of antigen presentation-related genes 2. Further stimulate the interferon signaling pathway to induce the tumor intrinsic immunogenicity 3. Combining LSD1 inhibitors with ICB therapy effectively enhances the immune response against treatment-resistant tumors	35, 37, 103
Bomedemstat (Phase 1/2)	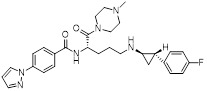	Irreversible LSD1 inhibitor	1. Increase the expression of MHC-I on the tumor cell surface, enhancing the induction of MHC-I by IFN-γ 2. Inhibition of LSD1 can enhance the sensitivity of tumors to PD-1 inhibitory responses, promoting CD8+ T cell infiltration and achieving strong tumor growth inhibition	36
Compound 3s	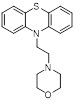	Reversible LSD1 inhibitor	1. Down-regulated PD-L1 on tumor cell surface 2. Promote T cell killing ability	106
SP2509	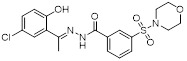	Reversible LSD1 inhibitor	1. Promote the increase of CD8+ T cell infiltration 2. Combined with anti-PD-(L)1 therapy, it can overcome tumor immune evasion and significantly inhibit tumor growth 3. Reduce the level of Ki-67 in tumors 4. Promote FAS ligands on CAR T cells to bind to FAS receptors of tumor cells and enhance their killing ability.	99, 100, 148, 158
SP-2577 (Phase 1/2)	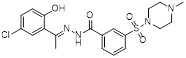	Reversible LSD1 inhibitor	1. Promote T cell infiltration	144
Phenelzine	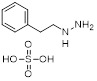	Irreversible MAO inhibitor	1. Combined with chemotherapy to reduce tumor volume and eliminate mesenchymal features 2. Promote the tumor-killing immune response of M1 macrophages	78
Tranylcypromine (Phase 1/2)	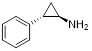	Irreversible MAO inhibitor	1. The combination with PD-1 antibody significantly inhibited tumor growth and lung metastasis 2. Reduce the level of Ki-67 in tumors 3. Promote CD8+ T cell infiltration	99
Compound 6x	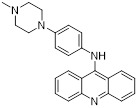	Reversible LSD1 inhibitor	1. Down-regulated PD-L1 on tumor cell surface 2. Promote T cell killing ability	107
Compound 5ac	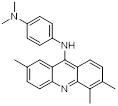	Reversible LSD1 inhibitor	1. Inhibit the stemness of tumor cells 2. Down-regulated PD-L1 on tumor cell surface 3. Promote T cell killing ability	105
Compound Z-1	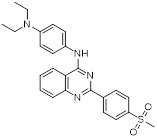	Reversible LSD1 inhibitor	1. Down-regulated PD-L1 on tumor cell surface 2. Promote T cell killing ability	108
GSK2879552 (Phase 1/2 terminated)	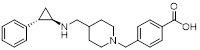	Irreversible LSD1 inhibitor	1. Prolong responses to PD-1 blockade 2. Promote CD8+ T cell infiltration 3. Sustain T cell invigoration	102, 160

## Abbreviations

AML: Acute myeloid leukemia
APCs: Antigen-presenting cells
CoREST: REST corepressor 1
CAFs: Cancer-associated fibroblasts
C/EBPα: CCAAT/enhancer-binding protein α
CML: Chronic myelogenous leukemia
CD141Hi cDCs: CD141Hi conventional dendritic cells
CRC: Colorectal cancer
CC: Cervical cancer
DNMT1: DNA methyltransferase 1
DCs: Dendritic cells
DIPG: Diffuse intrinsic pontine glioma
dsRNA: Double-stranded RNA
E2F1: E2F transcription factor 1
EMT: Epithelial-mesenchymal transition
FAD: Flavin adenine dinucleotide
GC: Gastric cancer
HDAC1/2: Histone deacetylase 1 and 2
H3K4me1/me2: Monomethyl and dimethyl histone H3 lysine 4
HIF-1α: Hypoxia-inducible factor-1
HNSCC: Head and neck squamous cell carcinoma
HCC: Hepatocellular carcinoma
ICB: Immune checkpoint blocking
IL-6: Interleukin-6
IL-4: Interleukin-4
IRF8: Interferon regulatory factor-8
IFN: Interferon
ISGs: IFN-stimulating genes
LSD1: Histone lysine-specific demethylase 1
LUAD: Lung adenocarcinoma
MHC: Major histocompatibility complex
MDS: Myelodysplastic syndromes
MSCs: Mesenchymal stem cells
MM: Multiple myeloma
MEF2D: Myocyte enhancer factor 2D
NK: Natural killer cells
NSCLC: Non-small cell lung cancer
OCCC: Ovarian clear cell carcinoma
OSCC: Oral squamous cell carcinoma
PRRs: Pattern recognition receptors
PC: Prostate cancer
RISC: RNA-induced silencing complex
STAT3: Signal transducer and activator of transcription 3
SCLC: Small cell lung cancer
SCCOHT: Small cell carcinoma of the ovary hypercalcemic type
TME: Tumor microenvironment
TAM: Tumor-associated macrophages
TNBC: Triple-negative breast cancer
TCR: T cell receptor
TNBC: Triple negative breast cancer
TGF-β: Transforming growth factor-β
Treg: Regulatory T
TNF-α: Tumor necrosis factor-α
ULBPs: UL16-binding protein
VEGF: Vascular endothelial growth factor
NE: Neuroendocrine
